# MAPK15 upregulation promotes cell proliferation and prevents DNA damage in male germ cell tumors

**DOI:** 10.18632/oncotarget.8044

**Published:** 2016-03-14

**Authors:** Matteo Rossi, David Colecchia, Gennaro Ilardi, Mario Acunzo, Giovanni Nigita, Federica Sasdelli, Angela Celetti, Angela Strambi, Stefania Staibano, Carlo Maria Croce, Mario Chiariello

**Affiliations:** ^1^ Istituto Toscano Tumori (ITT), Core Research Laboratory (CRL), AOU Senese, Siena, Italy; ^2^ Istituto di Fisiologia Clinica (IFC), Consiglio Nazionale delle Ricerche (CNR), Siena, Italy; ^3^ Dipartimento di Scienze Biomediche Avanzate, Università di Napoli “Federico II”, Napoli, Italy; ^4^ Department of Molecular Virology, Immunology and Medical Genetics, Comprehensive Cancer Center, The Ohio State University, Columbus OH, USA; ^5^ Istituto di Endocrinologia e Oncologia Sperimentale “G. Salvatore”, CNR, Napoli, Italy

**Keywords:** MAP kinases, autophagy, p53, DNA damage, embryonal carcinomas

## Abstract

Germ cell tumors (GCT) are the most common malignancies in males between 15 and 35 years of age. Despite the high cure rate, achieved through chemotherapy and/or surgery, the molecular basis of GCT etiology is still largely obscure. Here, we show a positive correlation between MAPK15 (ERK8; ERK7) expression and specific GCT subtypes, with the highest levels found in the aggressive embryonal carcinomas (EC). Indeed, in corresponding cellular models for EC, MAPK15 enhanced tumorigenicity *in vivo* and promoted cell proliferation *in vitro*, supporting a role for this kinase in human GCT. At molecular level, we demonstrated that endogenous MAPK15 is necessary to sustain cell cycle progression of EC cells, by limiting p53 activation and preventing the triggering of p53-dependent mechanisms resulting in cell cycle arrest.

To understand MAPK15-dependent mechanisms impinging on p53 activation, we demonstrate that this kinase efficiently protects cells from DNA damage. Moreover, we show that the ability of MAPK15 to control the autophagic process is necessary for basal management of DNA damage and for tumor formation controlled by the kinase.

In conclusion, our findings suggest that MAPK15 overexpression may contribute to the malignant transformation of germ cells by controlling a “stress support” autophagic pathway, able to prevent DNA damage and the consequent activation of the p53 tumor suppressor. Moreover, in light of these results, MAPK15-specific inhibitors might represent new tools to enhance the therapeutic index of cytotoxic therapy in GCT treatment, and to increase the sensitivity to DNA-damaging drugs in other chemotherapy-resistant human tumors.

## INTRODUCTION

Germ cell tumors (GCT) are the most common malignancies in males between the ages of 15 and 35 years [1–4], covering approximately 1% of all cancers [5] in men. Epidemiologic studies in USA and Europe have revealed an increasing trend in the incidence of GCT during the past several decades [6,7], whose reasons are still unclear. Interestingly, they represent only 0.1% of cancer-related deaths in men, thanks to the high cure rate (>90%), achieved through chemotherapy and/or surgery [5]. Consequently, GCT have always been studied in the attempt to understand their extraordinary sensitivity to chemotherapeutic drugs, to take advantage of this information for the therapeutic approach to other tumors. Still, the pathogenesis of germ cell neoplasms remains largely obscure [5].

Epidemiologically, clinically and histologically, distinct groups of GCT can be defined [8,9]: seminomatous tumors can be divided in seminomas and spermatocytic seminomas, whereas non-seminomatous germ cell tumors (NSGCT) include embryonal carcinomas (EC), choriocarcinomas, teratomas and yolk sac tumors [5]. GCT often arise in the form of mixed tumors, comprising a mixture of seminomatous and non-seminomatous components, with two or more histological subtypes present in variable proportions and distributed haphazardly throughout the tumor [5]. Such mixed GCT account for about 60% of cases [10,11]. The most common components in order of frequency are EC, teratoma, yolk sac tumor and seminoma [12].

Pure EC represent 3-4% of GCT and are the second most common single-cell-type GCT after seminomas [13]. As a component, however, they are present in more than 80% of mixed GCT [10,11]. EC is allegedly the most malignant form of testicular cancer: vascular and lymphatic invasion and infiltration of paratesticular tissue and the epididymis are common [13] and retroperitoneal lymph nodes or distant metastases at the time of diagnosis are reported in approximately two thirds of cases [14].

Mitogen-activated protein kinase 15 (MAPK15; ERK8; ERK7) is the last identified member of the MAP kinase family [15], whose activity and overexpression have been recently correlated to transformation of human colon cancer cells [16] and copy number gain in human gastric cancer [17], respectively. Interestingly, its activity is modulated by clinically relevant human oncogenes [18,19], whereas, in turn, it controls the activity of several nuclear receptors [20–22]. Furthermore, MAPK15 is required for proliferating cell nuclear antigen (PCNA) protein stability and, thus, for the maintenance of genomic integrity [23]. Also, it strongly affects telomerase activity [24], making this kinase an important player in the mechanisms contributing to bypass replicative senescence and to immortalize cells. Nonetheless, data linking MAPK15 to specific human tumors are still very limited.

MAPK15 is also a potent regulator of autophagy [25] and, in its absence, both basal and starvation-induced autophagy are significantly impaired [25]. Indeed, MAPK15 is a key kinase transducing signals elicited by serum and aminoacid starvation: in these conditions, it mediates the stress-induced increase of the autophagic response [25] and leads to inhibition of protein secretion [26]. Interestingly, we have recently demonstrated that MAPK15 mediates BCR-ABL-induced autophagy and regulates oncogene-dependent cell proliferation and tumor formation [19].

In the present study, we found that MAPK15 is overexpressed in specific subsets of GCT, i.e. seminomas and embryonal carcinomas (EC). Moreover, in a cellular model for EC, namely NTera2/D1 cells, we show that it protects from accumulation of reactive oxygen species (ROS) and DNA damage, therefore preventing p53 activation and p53-mediated cell cycle arrest, and thus favoring proliferation and tumorigenicity of these GCT-derived cell lines. Ultimately, we provide evidences that MAPK15-dependent autophagy is necessary for the control of DNA damage and for tumor formation in this context.

## RESULTS

### *Mapk15* is highly expressed in the male gonads and its upregulation is a conserved trait throughout evolution

**Figure 1:**
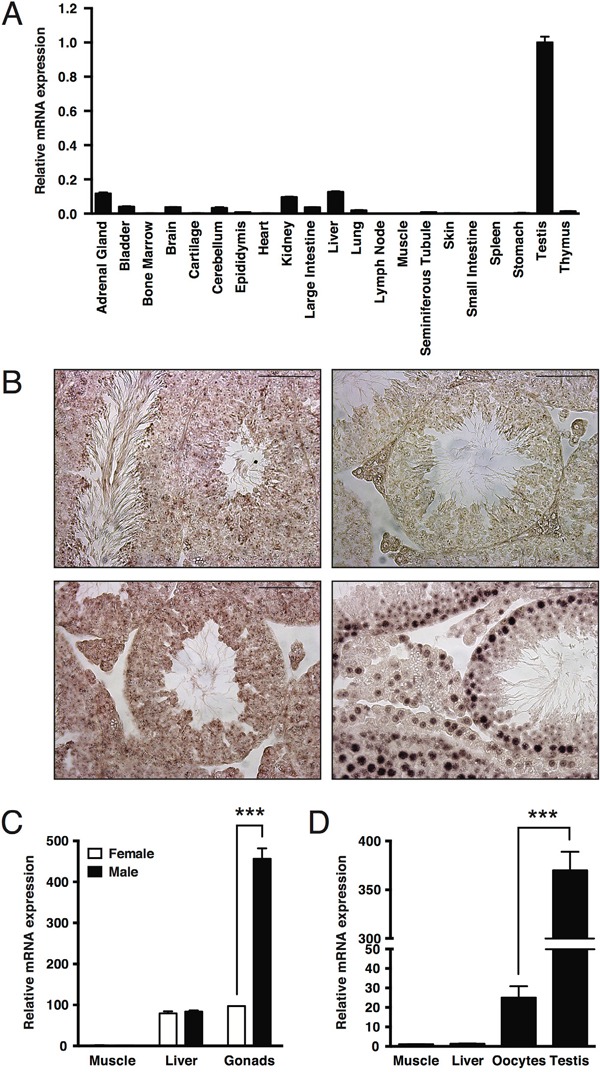
Elevated expression of MAPK15 in male gonads is a conserved trait in mouse and X. laevis **A.** Expression levels of *Mapk15* in a panel of tissues from adult CD1 male mice, assessed by quantitative real-time PCR. **B.** *In-situ* hybridization (ISH) on paraffin-embedded adult CD1 mouse testis. Sections were probed with a *Mapk15*-specific LNA probe (upper left), with a LNA probe against sense miR159 from Arabidopsis thaliana (upper right) as negative control and with LNA probes against *Actb* (lower left) and *U6 snRNA* (lower right) as positive controls. Scale bars correspond to 25 μm. **C.** Expression levels of *Mapk15* in male and female gonads from adult CD1 mice, assessed by quantitative real-time PCR. **D.** Expression levels of *mapk15* in testis and oocytes from adult X. laevis, assessed by quantitative real-time PCR. Each bar represents the average ± SEM of three PCR replicates. Significance (p value) was assessed by Student's t test. Asterisks were attributed as follows: ***p<0.001.

To get insights into important functions of the MAPK15 gene in specific human tissues, we performed quantitative real-time PCR on a panel of adult CD1 male mouse organs, which revealed that expression of the *Mapk15* gene is highest in the testis as compared to any other mouse tissue (Fig. 1A). We confirmed the presence of *Mapk15* mRNA in the adult mouse testis by *in-situ* hybridization (ISH) with a *Mapk15*-specific LNA probe (Fig. 1B, upper left panel). A probe against sense miR159 from Arabidopsis thaliana was used as negative control (Fig. 1B, upper right), whereas probes against mouse *ACTB* (Fig. 1B, lower left) and U6 snRNA (Fig. 1B, lower right) were used as positive controls. In order to ascertain whether *Mapk15* elevated expression in the gonads is present also in the female, we compared *Mapk15* mRNA levels in muscle, liver and gonads form adult male and female CD1 mice by real-time PCR. Expression in the testis was significantly higher than in the ovary (∼4.7-fold), whereas no difference was observed between sexes in the muscle and in the liver (Fig. 1C). Still, a bias in this observation might be introduced by the lower relative abundance of oocytes in the whole ovary compared to the abundance of male germ cells in the whole testis. Hence, we repeated the analysis with samples obtained from X. laevis, a system that can easily supply high amount of purified oocytes. Frog *mapk15* expression levels were ∼14.8-fold higher in male germ cells compared to oocytes (Fig. 1D), confirming the data obtained in the mouse, but suggesting that MAPK15 may have important functions also in female germ cells [27]. Supporting these evidences, analysis of expression data available on FlyBase (http://flybase.org) [28], a database of Drosophila genes and genomes, revealed that CG31703, the *MAPK15* ortholog in Drosophila melanogaster, was barely detectable or absent in the embryo and in early larval stages but gradually increased from larval stage L3, reaching its maximal expression in the adult male fly. Interestingly, CG31703 was not detectable in the adult female fly (Suppl. Fig. 1A) whereas the highest levels were observed in the adult male testis (Suppl. Fig. 1B).

Overall, the extremely high expression of *MAPK15* specifically in male gonads from different, evolutionary distant species, despite the extremely low conservation score of *MAPK15* throughout evolution [29], suggests its importance in male germ cell biology and, possibly, pathology.

### MAPK15 is overexpressed in the malignant components of male GCT

**Figure 2:**
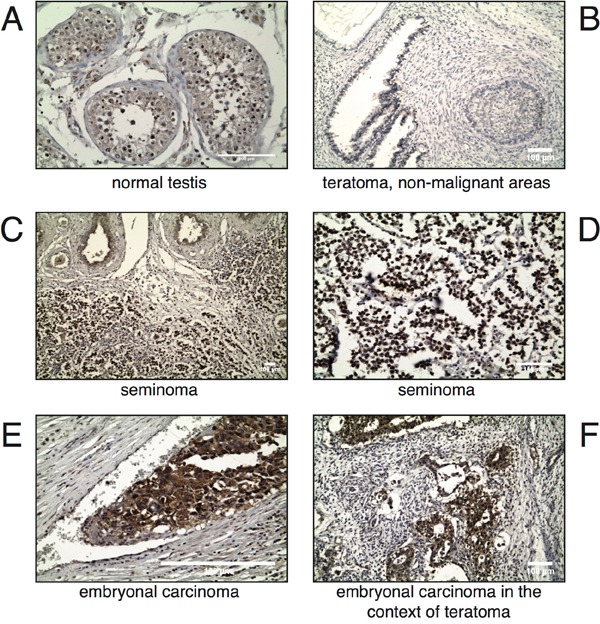
MAPK15 is differentially expressed in male germ cell tumors Immunohistochemistry (IHC) was performed on paraffin-embedded specimens of human origin using a MAPK15-specific antibody. Representative IHC images of archival samples from human normal testis **A.** teratoma, non-malignant areas **B.** seminoma **C, D.** embryonal carcinoma **E.** and embryonal carcinoma in a context of teratoma **F.** are shown. Scale bars correspond to 100 μm.

MAPK15 is involved in key biological processes, such as the maintenance of genomic integrity [23], the regulation of telomerase activity [24] and autophagy [19,25], that can lead, when deregulated, to cell transformation. Also, its interplay with *bona fide* human oncogenes is now acknowledged [15,16,18]. Still, very limited information is yet available regarding its expression and role in specific human tumors [16,17]. Based on these evidences, and on the aforementioned data demonstrating high mRNA expression of *MAPK15* in the testis, we hypothesized a possible role for this kinase also in testicular cancer. To investigate the involvement of the MAPK15 protein in GCT, its expression was assessed by immunohystochemistry (IHC) on a tissue array of various human specimens, and each neoplastic sample was compared to its normal counterpart. Interestingly, whereas MAPK15 was moderately overexpressed in all pure seminomas (Table 1), the analysis of non-seminomatous germ cell tumors revealed a more complex expression pattern. Indeed, MAPK15 was not detectable in non-malignant teratoma areas, was moderately expressed in the seminoma component, whereas was highly expressed in the malignant embryonal carcinoma (EC) component (Table 2). In figure 2, representative IHC images are shown. Based on these data, it is therefore plausible to hypothesize a contribution of MAPK15 to the pathogenesis of human male GCT, in particular EC.

**Table 1:** MAPK15 expression in human seminomatous germ cell tumors

Sample	Age (years)	Diagnosis	MAPK15 expression
1	43	Seminoma	++
2	25	Seminoma	++
3	20	Seminoma	++
4	38	Seminoma	++
5	15	Seminoma	+
6	46	Seminoma	++
7	47	Seminoma	++
8	26	Seminoma	+
9	17	Seminoma	++
10	30	Seminoma	++
11	42	Seminoma	++
12	37	Seminoma	++
13	39	Seminoma	++
14	34	Seminoma	+
15	35	Seminoma	++
16	37	Seminoma	++
17	32	Seminoma	++
18	42	Seminoma	++
19	41	Seminoma	++
20	33	Seminoma	+
21	39	Seminoma	++
22	39	Seminoma	++
23	37	Seminoma, anaplastic	+

**Table 2:** MAPK15 expression in human nonseminomatous germ cell tumors

Sample	Age (years)	Diagnosis	Histotype	MAPK15 expression		
				TE^1^	SE^2^	EC^3^
24	39	Non-seminoma	SE EC		++	+++
25	37	Non-seminoma	TE SE	0	++	
26	17	Non-seminoma	TE EC	0		+++
27	25	Non-seminoma	TE EC	0		+++
28	45	Non-seminoma	TE EC	0		+++
29	27	Non-seminoma	TE SE EC	0	++	+++
30	31	Non-seminoma	SE EC		++	+++
31	26	Non-seminoma	SE EC		++	+++
32	35	Non-seminoma	EC			+++
33	34	Non-seminoma	EC			+++
34	39	Non-seminoma	EC			+++
35	30	Non-seminoma	EC			+++
36	34	Non-seminoma	EC			+++
37	28	Non-seminoma	TE	0		
38	30	Non-seminoma	EC			+++

1 TE: Teratoma.

2 SE: Seminoma.

3 EC: Embryonal carcinoma

### MAPK15 affects the tumorigenicity of human GCT-derived cell lines

**Figure 3:**
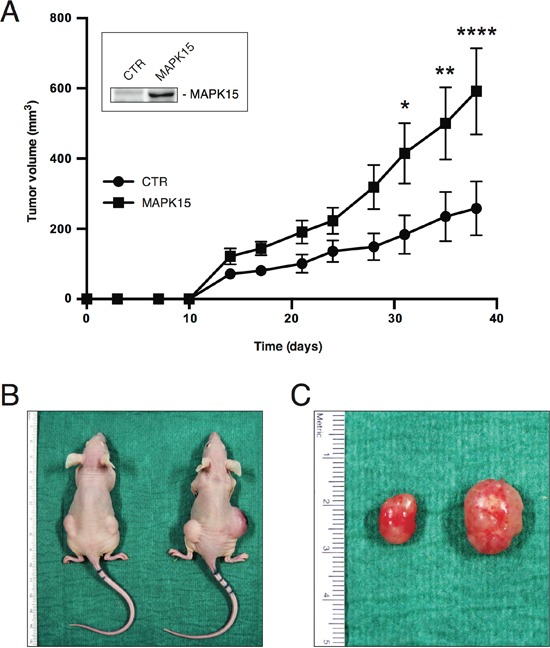
MAPK15 affects the tumorigenicity of human GCT-derived cell lines **A.** Growth curves of tumors generated by NTera2/D1 cells stably expressing MAKP15 or a control vector (pCEFL, CTR), after injection on the flank of athymic nude-Foxn1^nu^ mice. Each point represents the mean volume ± SEM of ten tumors. The experiment was performed in triplicate. Inset shows MAPK15 protein levels in the two stable cell lines before injection, determined using a MAPK15-specific antibody. **B.** Representative mice bearing NTera2/D1 pCEFL- (left mouse) or NTera2/D1 MAPK15-derived tumors (right mouse). **C.** Representative tumor specimen excised from a control xenograft (left) in comparison to its MAPK15-positive counterpart (right). Significance (p value) was assessed by Student's t test, comparing the CTR and MAPK15-overexpressing conditions for each time-point. Asterisks were attributed as follows: *p< 0.05, **p<0.01, ****p<0.0001.

To confirm, *in vivo*, a pro-tumorigenic role for MAPK15 overexpression in human EC, we took advantage of the best-characterized cell line derived from this kind of tumor, namely NTera2/D1 cells [30], and used it in classical xenograft experiments. We generated two stable cell lines from NTera2/D1, expressing MAPK15 (NTera2/D1_MAPK15) or a control vector (NTera2/D1_pCEFL), respectively (Fig. 3A, inset), to study the contribution of MAPK15 to the tumorigenicity of EC cells *in vivo*. To this aim, NTera2/D1_pCEFL and NTera2/D1_MAPK15 were inoculated in both flanks of athymic nude-Foxn1^nu/nu^ mice and tumor growth was monitored over a time span of ∼6 weeks. Tumor growth rates in the two experimental groups significantly diverged early on in the period of observation, and tumors generated by NTera2/D1_MAPK15 were on average ∼2.3-fold the size of the controls, at the experimental endpoint (Fig. 3A). Two representative mice are depicted in Figure 3B, bearing NTera2/D1_pCEFL- (left mouse) or NTera2/D1_MAPK15-derived tumors (right mouse), respectively. In Figure 3C, a representative tumor specimen excised from a control xenograft (left) is shown in comparison to its MAPK15-positive counterpart (right). These data support our observations on MAPK15 overexpression in human testicular malignancies (see Table 1, Table 2 and Fig. 2). Indeed, the more aggressive phenotype granted by MAPK15 overexpression to NTera2/D1 cells nicely fits with the evidence that MAKP15 levels positively correlate to germ cell tumor malignancy in patients, being this kinase downregulated in the more benign teratomas and upregulated in the malignant EC.

### MAPK15 promotes the growth of human GCT-derived cell lines

**Figure 4:**
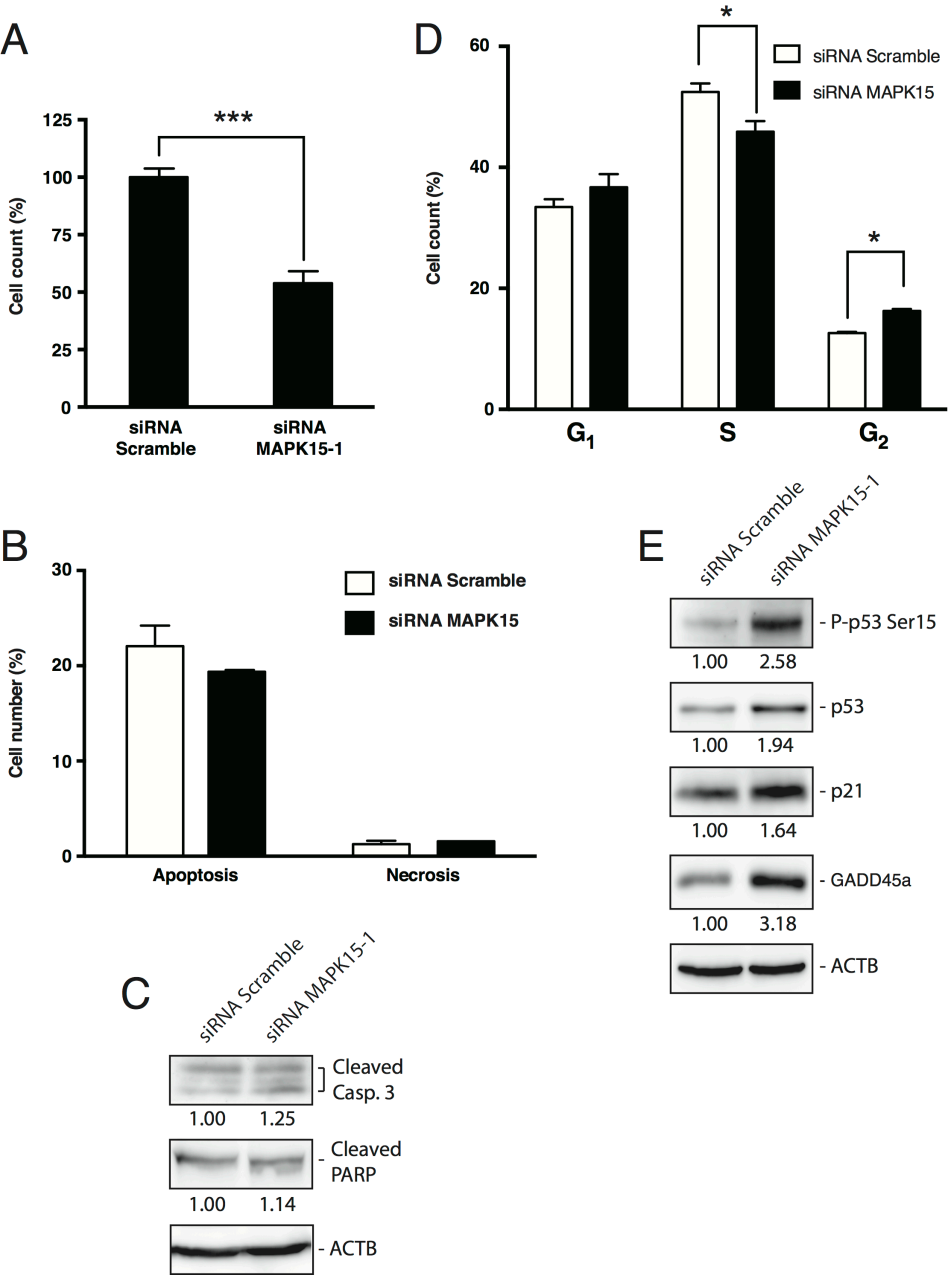
MAPK15 affects the growth of human GCT-derived cell lines **A.** Cell count of NTera2/D1 cells transfected with *MAPK15*-targeted siRNA or non-silencing (Scramble) siRNA, assessed by trypan blue exclusion assay 72 h post-transfection. The transfections were performed in triplicate and the resulting mean values ± SEM were expressed as a percentage of mean cell number normalized on the Scramble-transfected sample. **B.** Percentage of apoptotic and necrotic cells following RNA interference with siRNA specific for *MAPK15*, compared to a control transfected with non-silencing (Scramble) siRNA, as assessed by Annexin V staining 72 h post-transfection. **C.** Levels of apoptotic markers following *MAPK15* knockdown. Lysates from NTera2/D1 cells interfered for *MAPK15*, compared to a control transfected with Scramble siRNA, were probed with antibodies specific for cleaved Caspase-3 and cleaved PARP. ACTB (actin) was used as loading control and for normalization purposes. Intensitometric analysis was performed with ImageQuant TL software (GE Healthcare). **D.** Cell cycle analysis, performed by EdU staining, of NTera2/D1 cells interfered for *MAPK15*, compared to a control transfected with Scramble siRNA. The transfections and subsequent analyses were performed in triplicate and the results are expressed as the average ± SEM of the three biological replicates. **E.** Activation of the DNA damage response following *MAPK15* knockdown. Lysates from NTera2/D1 cells interfered for *MAPK15*, compared to a control transfected with Scramble siRNA, were probed with antibodies specific for phospho-p53 (Ser15), total p53, CDKN1A/p21 and GADD45a. ACTB (actin) was used as loading control and for normalization purposes. Intensitometric analysis was performed with ImageQuant TL software (GE Healthcare). Significance (p value) was assessed by Student's t test. Asterisks were attributed as follows: *p< 0.05, ***p<0.001.

MAPK15 is therefore able to promote tumor growth, *in vivo*, in GCT models. In order to elucidate the mechanisms by which this kinase may contribute to the tumorigenicity and proliferation of EC cells, we first investigated the effect of endogenous MAPK15 knockdown [22,25] in NTera2/D1 cells. Transfection of NTera2/D1 cells with a MAPK15-specific siRNA (Suppl. Fig. 2) led to ∼50% decrease in cell counts compared to the scrambled silenced control, as scored by trypan blue exclusion assay, performed 72 h post-transfection with MAPK15-specific siRNA (Fig. 4A). Notably, this effect was not due to a decrease in cell viability. Indeed, we did not observe significant changes in the number of apoptotic and necrotic cells (Fig. 4B), as scored by Annexin V staining, and the change in the levels of cleaved Caspase-3 and cleaved PARP, two well-established apoptotic markers [31], was negligible (Fig. 4C), as also already demonstrated in HeLa cells [19]. Conversely, transfection with MAPK15-specific siRNA led to a significant reduction in the percentage of cells in S phase and to a concomitant increase in the percentage of cells in G_2_ phase (Fig. 4D), as a possible consequence of p53 activation [31–33]. Indeed, MAPK15 knockdown triggered the phosphorylation of p53 on Ser15 [31], along with p53 protein stabilization, and upregulation of CDKN1A/p21 and GADD45a, two *bona fide* p53 target genes responsible for p53-mediated cell cycle arrest [31,34] (Fig. 4E). Such results were also confirmed and further expanded in Suppl. Fig. 3A and Suppl. Fig. 3B by using a different MAPK15-specific siRNA and a different embryonic carcinoma cell line (N2102). Altogether, these evidences point out a role for MAPK15 in sustaining cell cycle progression of GCT-derived cell lines.

### MAPK15 prevents the accumulation of DNA damage in GCT-derived human cell lines

**Figure 5:**
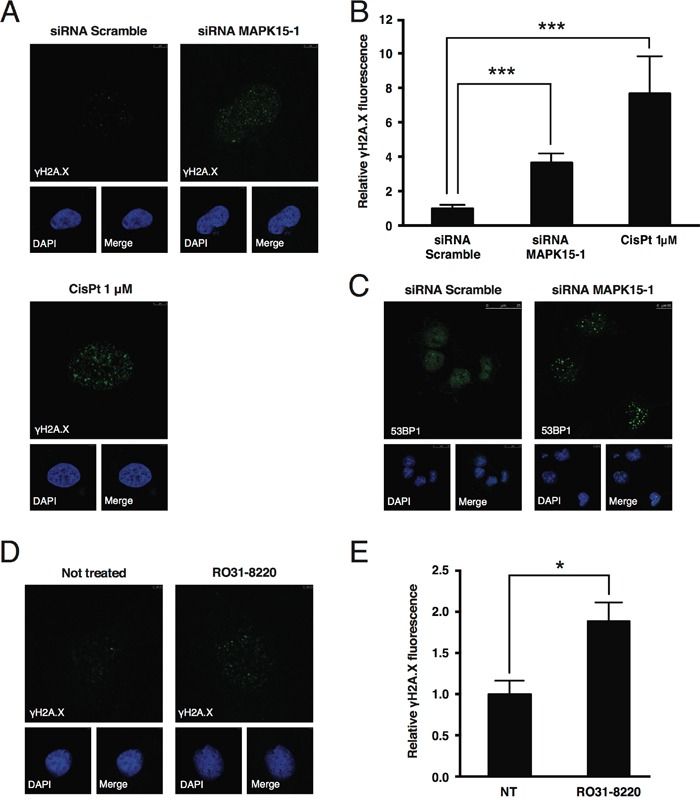
MAPK15 protects the genomic integrity of human GCT-derived cell lines **A.** Representative confocal microscopy images showing DNA damage, scored as accumulation of nuclear γH2A.X foci, in NTera2/D1 cells following transfection with non-silencing (Scramble) siRNA (upper left panel) or siRNA specific for *MAPK15* (upper right panel), compared to a positive control treated with 1 μM Cis-Pt (lower panel). Cells were stained with anti-γH2A.X primary antibody and Alexa Fluor 488-conjugated secondary antibody. Nuclei were stained with DAPI. **B.** Intensitometric analysis of nuclear γH2A.X fluorescence, identified as green fluorescence colocalizing with DAPI. Total nuclear green fluorescent signal was quantified in five representative fields using Volocity software. A total of approximately 100 cells were analyzed for each sample. **C.** Representative confocal microscopy images showing DNA damage, scored as accumulation of nuclear 53BP1 foci, in NTera2/D1 cells following transfection with Scramble siRNA (left panel) or siRNA specific for *MAPK15* (right panel). Cells were stained with anti-53BP1 primary antibody and Alexa Fluor 488-conjugated secondary antibody. Nuclei were stained with DAPI. **D.** Representative confocal microscopy images showing DNA damage, scored as accumulation of nuclear γH2A.X foci, in NTera2/D1 cells following O/N treatment with the MAPK15 inhibitor RO31-8220 at the concentration of 100 nM (right panel), compared to an untreated control (left panel). Cells were stained with anti-γH2A.X primary antibody and Alexa Fluor 488-conjugated secondary antibody. Nuclei were stained with DAPI. **E.** Intensitometric analysis of nuclear γH2A.X fluorescence, identified as green fluorescence colocalizing with DAPI, in NTera2/D1 cells following O/N treatment with the MAPK15 inhibitor RO31-8220 at the concentration of 100 nM, compared to an untreated control (NT). Total nuclear green fluorescent signal was quantified in five representative fields using Volocity software. Bars represent the average ± SEM of five representative microscopy fields. Significance (p value) was assessed by Student's t test. Asterisks were attributed as follows: *p<0.05, ***p<0.001.

Available data indicate that MAPK15 contributes, through various mechanisms, to the maintenance of genomic stability [23,24,35]. We, therefore, speculated that MAPK15 knockdown might negatively impact on cell growth by increasing genomic instability, thus triggering, as a consequence, the p53-dependent DNA damage response (DDR) pathway. This, in turn, leads to cell cycle arrest and eventually to DNA repair or, alternatively, to cell death, depending on the severity of the damage [31]. In line with our hypothesis, even in absence of extrinsic genotoxic stress, we observed a ∼4-fold increase in DNA damage, scored as the intensity of nuclear phospho-Ser139 H2AX (γH2A.X) signal [36], in NTera2/D1 cells 72 h after transfection with a MAPK15-specific siRNA (Fig. 5A and B). Likewise, in the same experimental conditions, another well-established DNA damage marker, namely 53BP1 [37], relocalized to DNA damage foci (Fig. 5C). Concurrently, phosphorylation of ATR and CHK2 increased [38] (Suppl. Fig. 4). These results were also confirmed and further expanded in Suppl. Fig. 5 by using a different MAPK15-specific siRNA (MAPK15-2) and a different embryonic carcinoma cell line (N2102).

To confirm MAPK15 involvement in the control of DNA damage in NTera2/D1 through a different approach, we treated these cells with the MAPK15 inhibitor RO31-8220 [24,25,39], which, in line with our expectations, determined an increase in γH2A.X staining (Fig. 5D and E).

Altogether, these evidences indicate a role for MAPK15 in promoting GCT-derived cell lines growth, by reducing the accumulation of DNA damage and the consequent activation of DDR-triggered cell cycle arrest.

### MAPK15-induced autophagy is essential to prevent DNA damage accumulation in GCT-derived cell lines

**Figure 6:**
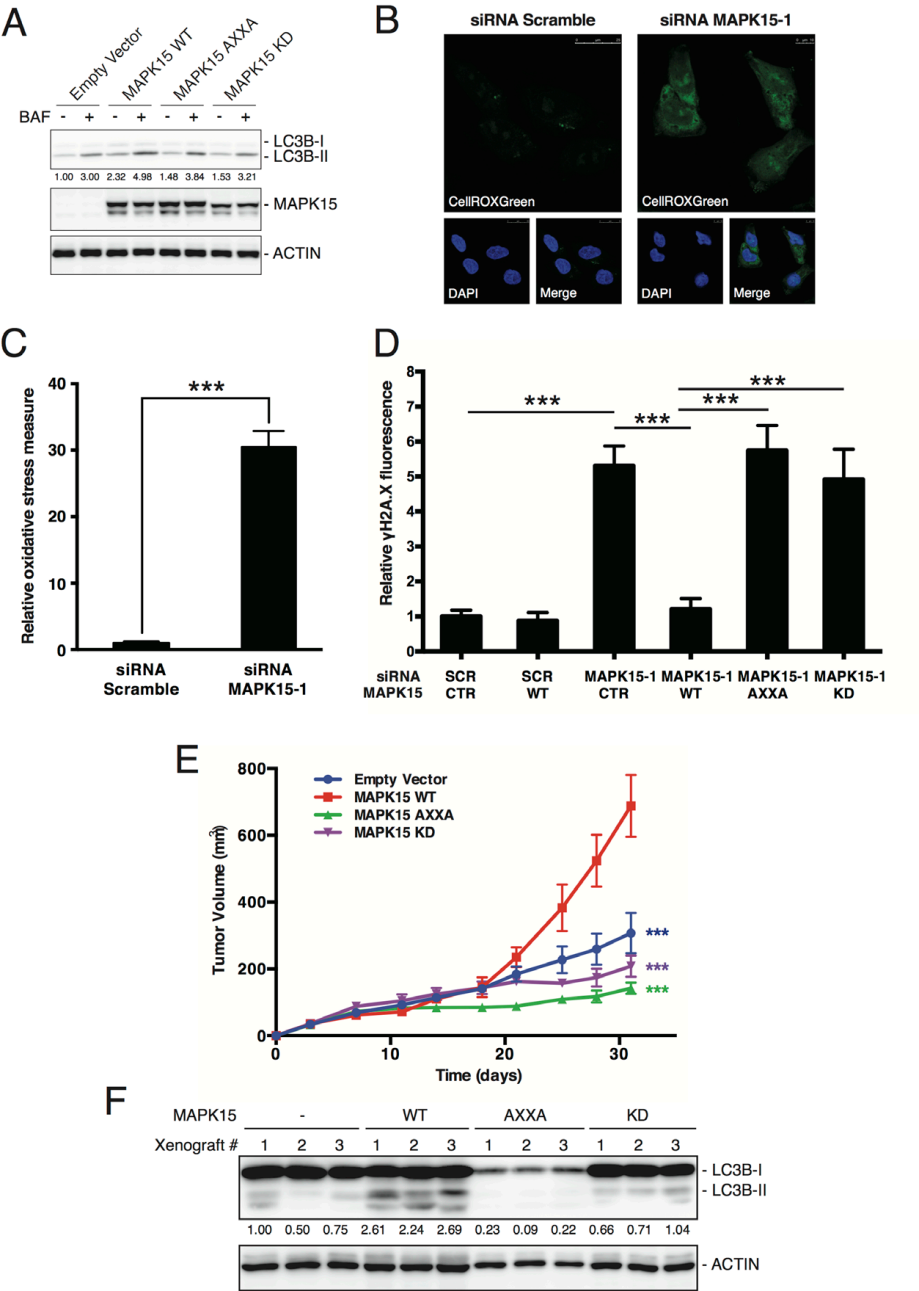
MAKP15-induced autophagy is necessary for the maintenance of genomic integrity in human GCT-derived cell lines **A.** Autophagic flux, in the absence or presence of bafilomycin A_1_ (BAF), was evaluated in NTera2/D1 cells stably expressing wild-type (WT), Y340A-I343A autophagy-deficient (AXXA) or kinase-dead (KD) MAPK15. Control sample (CTR) was transfected with the empty vector. Lysates were probed with antibodies specific for LC3B. ACTB (actin) was used as loading control and for normalization purposes. **B.** Representative confocal microscopy images showing accumulation of oxidative stress, in NTera2/D1 cells following transfection with Scramble siRNA (left panel) or siRNA specific for *MAPK15* (right panel). Cells were stained with the reactive oxygen species (ROS)-specific green fluorescent probe CellROXGreen. Nuclei were stained with DAPI. **C.** Intensitometric analysis of the accumulation of oxidative stress, identified as CellROXGreen fluorescence. Total green fluorescent signal was quantified in five representative fields using Volocity software. **D.** Intensitometric analysis of nuclear γH2A.X fluorescence, identified as green fluorescence colocalizing with DAPI, in NTera2/D1 cells transfected with Scramble siRNA or siRNA specific for *MAPK15*, followed by rescue with ectopically expressed wild-type (WT), Y340A-I343A autophagy-deficient (AXXA) or kinase-dead (KD) MAPK15. Control sample (CTR) is transfected with the empty vector. Total nuclear green fluorescent signal was quantified in five representative fields using Volocity software. Bars represent the average ± SEM of five representative microscopy fields. A total of approximately 100 cells were analyzed for each sample. **E.** Growth curves of tumors generated by NTera2/D1 cells stably expressing empty vector, MAPK15 WT, MAPK15 AXXA and MAPK15 KD, after injection on the flank of athymic nude-Foxn1^nu^ mice. Each point represents the mean volume ± SEM of 16 tumors. Student's t test for tumor volume at the time point of 31 days comparing each MAPK15 mutant xenograft group (MAPK15_AXXA and MAPK15_KD) with MAPK15_WT xenograft group resulted in a *p* value significance lower than 0.001 (***). Comparison between empty vector group and MAPK15_WT resulted in a *p* value significance lower than 0.001 (***). **F.** Autophagic activity was evaluated in xenograft tumors derived from NTera2/D1 cells stably expressing wild-type (WT), Y340A-I343A autophagy-deficient (AXXA) or kinase-dead (KD) MAPK15. Control sample (−) was transfected with the empty vector. Tumors were recovered and analyzed at the endpoint (31 days; panel E). Lysates were probed with antibodies specific for LC3B. ACTB (actin) was used as loading control and for normalization purposes. Significance (p value) was assessed by Student's t test. Asterisks were attributed as follows: ***p<0.001.

We already demonstrated that MAPK15 is a key regulator of autophagy, able to induce this process both in basal conditions and in response to stress [19,25,40]. Notably, autophagy is a fundamental contributor to genomic stability, exerting this role through multiple mechanisms aimed to maintain cellular homeostasis, e.g. degradation of dysfunctional mitochondria and toxic protein aggregates and removal of micronuclei and damaged nuclear parts [41]. Indeed, also in NTera2/D1, our model system for EC, pharmacological inhibition of autophagy led to a significant increase γH2A.X signal (Suppl. Fig. 6). We therefore reasoned that the ability of MAPK15 to stimulate autophagy might represent a novel mechanism by which this kinase contributed to the maintenance of genomic stability. In order to verify this hypothesis, we first tested whether MAPK15 controls autophagy in EC cell lines. Indeed, knockdown of MAPK15 in NTera2/D1 cells led to an accumulation of LC3-positive autophagic vesicles (Suppl. Fig. 7A). However, this effect may be indicative of either stimulation or inhibition of the autophagic process [42]. To discriminate between these two opposite effects, we monitored the autophagic flux by treating NTera2/D1 cells, transfected either with scramble or MAPK15-specific siRNA, with bafilomycin A_1_, an inhibitor of lysosomal acidification that prevents lysosomal degradation of autophagosome-associated LC3B and SQSTM1/p62 [42]. Following bafilomycin A_1_ treatment, we observed no accumulation of LC3B-II upon MAPK15 knockdown, whereas SQSTM1/p62 was stabilized (Suppl. Fig. 7B). Also, a specific autophagy-impaired mutant, MAPK15_AXXA (Y340A-I343A), which is no longer able to bind LC3B family members and, consequently, behaves as a dominant negative protein for autophagy [19,25], failed to stimulate autophagy, similarly to the MAPK15 kinase-dead mutant (Figure 6A). These results clearly indicate that MAPK15 controls the autophagic process in NTera2/D1 cells, and that high levels of MAPK15 are essential in sustaining basal autophagy, in these cells.

The inhibition of autophagy is often associated to the accumulation of reactive oxygen species (ROS) [43,44]. In line with these evidences, MAPK15 knockdown in EC cells dramatically increased oxidative stress (Fig. 6B and C), which may contribute to the accumulation of DNA damage that we observed in NTera2/D1 cells lacking MAPK15 expression. We confirmed the importance of MAPK15-induced autophagy in preventing DNA damage in EC cells by rescuing siRNA-depleted MAPK15 expression in NTera2/D1, with wild-type, autophagy-deficient and kinase-dead mutants (Suppl. Fig. 8). Notably, wild-type MAPK15, ectopically expressed in MAPK15-silenced cells, was able to reduce DNA damage to background levels (Figure 6D). Conversely, the specific autophagy-impaired mutant (MAPK15_AXXA) failed in this task (Fig. 6D). Interestingly, the kinase-dead mutant of MAPK15 (MAPK15_KD) also failed to rescue NTera2/D1 cells from DNA damage caused by MAPK15 knockdown (Fig. 6D), further supporting the evidence that MAPK15 kinase activity is essential for the induction of autophagy [25]. Interestingly, the autophagic mechanism described herein appears to be coexistent with MAPK15-dependent PCNA stabilization [23] in these specific cells as interfering with MAPK15 expression by specific siRNA led to a decrease in PCNA protein levels (Suppl. Fig. 9).

Ultimately, we reasoned that, if autophagy represents an important consequence of MAPK15 overexpression for tumorigenesis, then the autophagy-deficient MAPK15_AXXA mutant would confer no advantage for tumor growth in xenograft assays. Therefore, to evaluate the role of autophagy controlled by MAPK15 in *in vivo* tumor formation by NTera2/D1, we injected these cells stably expressing empty vector and WT, AXXA and KD MAPK15 mutants, in athymic nude-Foxn1^nu/nu^ mice and monitored their tumor growth over a time span of approximately 4 weeks. At the endpoint of observations, the MAPK15_AXXA mutant and the MAPK15_KD mutant significantly impaired NTera2/D1-dependent tumor growth, as compared to MAPK15_WT (Figure 6E). As a control, the MAPK15_AXXA mutant (as well as the kinase-dead mutant) strongly reduced autophagy in xenograft tumors, as scored by LC3B western blot assay (Fig. 6F). Altogether, these data show that the ability of MAPK15 to control the autophagic process is necessary for mediating its oncogenic effects.

Overall, our work therefore demonstrate a specific role for MAPK15-dependent autophagy in controlling DNA damage in NTera2/D1, possibly repressing p53-dependent mechanisms impinging on cell cycle progression of these EC-derived cells.

## DISCUSSION

The long-standing interest of reproductive sciences in the biology and physiology of male gamete generation has now led to a deeper understanding of the cellular processes involved in spermatogenesis, allowing for the identification of many previously overlooked genes playing important roles in this biological process. In particular, in most species, male gametes undergo a strong DNA-damage-dependent selection process, because, due to the high number of germ cell divisions taking place during sperm production, this process offers “high” mutational possibilities and, therefore, more “quality control” mechanisms are needed [45]. Based on our data, MAPK15 might contribute to the faithful transmission of genetic information to the offspring, by maintaining genomic stability. In this context, cancer is intimately associated with DNA damage at multiple levels. Unrepaired DNA damage may cause mutations or chromosomal aberrations contributing to malignant transformation, whereas DNA-damaging therapies are the major non-surgical treatment modalities currently used in oncology [46]. Indeed, genes whose products participate in DDR are commonly deregulated or inactivated in tumors [46,47], and defects in DNA damage recognition, signaling and/or downstream responses allow the genetically unstable cancer cells to survive, proliferate and acquire even more genetic instability [48].

**Figure 7:**
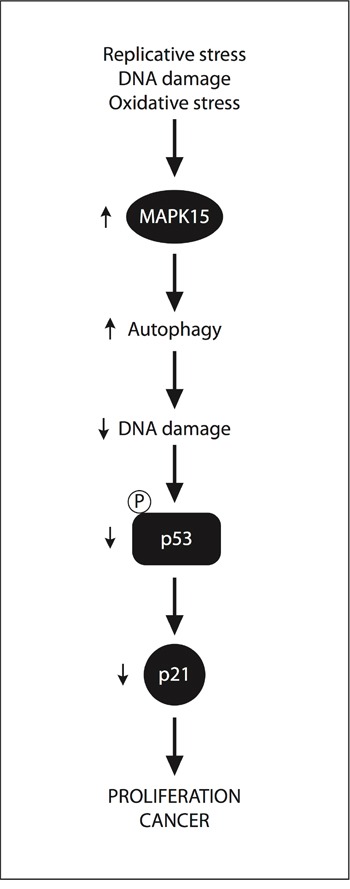
A “non-oncogene addition” model for MAPK15 involvement in GCT Specific forms of the “stress phenotype” in cancer (replicative stress, DNA damage stress and oxidative stress) [57] stimulate the expression and/or activity of the MAPK15 protein. The ability of MAPK15 to stimulate autophagy may, therefore, control DNA damage and p53-dependent responses (i.e., CDKN1A/p21 expression). As a consequence p53 does not effectively exert its control on cell cycle progression, thereby promoting cell proliferation and cancer.

In contrast to other solid tumors, p53 not only is expressed at higher than normal levels in most GCT, but is also rarely mutated (< 3%) or functionally inactivated [49,50]. This legacy of their origin from germ cells, which readily undergo apoptosis rather than risking unfaithful transmission of their genetic information [45], contributes to the exceptionally high sensitivity of GCT to DNA-damaging agents [51]. This characteristic allows the overall management of GCT through pharmacological and/or surgical treatment to results in a 90% to 95% cure rate. Nevertheless, resistance to chemotherapy may occur, especially in EC, enhanced DNA repair capacity being among the main drivers [52]. Here, we propose MAPK15 upregulation as a new mechanism by which GCT cells may restrain DDR-mediated p53 activation, thus promoting their own growth and tumorigenicity (Fig. 7).

To our surprise, depletion of MAPK15 strongly affected both NTera2/D1 cell proliferation and their growth as xenograft tumors without inducing appreciable apoptosis in these cells. Still, Attardi and colleagues [53] have already shown that inhibition of autophagy does not affect cell cycle arrest in response to DNA damage, whereas it is important for robust p53-dependent apoptosis triggered by DNA damage. Our hypothesis is, therefore, that MAPK15 depletion, while activating p53, impedes p53-dependent autophagy, in turn interfering with the full spectrum of p53-dependent cellular responses and, in particular, preventing its apoptotic program. In this context, the possibility arises that MAPK15 might specifically contribute to the p53-dependent apoptotic program, and we plan to further investigate this issue in the future.

Interestingly enough, MAPK15 has also already been proposed to function epistatically to p53 in the ribosome surveillance response and an increase in p53 levels correlated to induced *MAPK15* expression [54]. Hence, following cytotoxic stress, MAPK15 might act both as a downstream effector of p53, activating pathways involved in damage protection and recovery (e.g. autophagy [25]), and as a negative feedback mechanism on p53 itself [55]. Indeed, MAPK15, by promoting PCNA stabilization [23] and preventing ROS generation (this manuscript), might help in preventing DNA damage, thus contributing to shut down the DDR.

In this context, we believe it is important to underline that MAPK15 is able to prevent PCNA degradation by directly binding this protein and inhibiting its interaction with HDM2, without affecting the protein levels of this important E3 ligase, in mammary [23] as well as in EC cells (Suppl. Fig. 9). Therefore, although this mechanism can surely participate to protecting genomic integrity, the effect of MAPK15 on p53 stability cannot be mediated by direct MAPK15-dependent regulation of HDM2, a reported p53 E3 ligase [55]. On the contrary, we provide evidences that MAPK15 depletion by itself causes an increase in cellular ROS, an important cause of DNA damage, and that MAPK15-dependent autophagy is required for preventing double-strand breaks in EC cells.

Tumor cells constantly experience cellular stresses (e.g. replication, DNA damage and oxidative stress) not experienced by normal cells and, therefore, cancer cells are more dependent on stress support pathways (e.g. DDR and autophagy) for their survival [56]. Hence, based on the concept of “non-oncogene addition”, the tumorigenic state strongly depends on the activities of a wide variety of genes and pathways, many of which are not inherently oncogenic themselves [57], and such dependency can be exploited therapeutically through both stress sensitization and stress overload [56]. In light of our findings, MAPK15-specific inhibitors might, therefore, represent new tools to enhance the therapeutic index of cytotoxic therapy in GCT treatment and to increase sensitivity to DNA-damaging agents in chemotherapy-resistant GCT subsets and in other human tumors with acquired or intrinsic resistance.

Although additional work will be necessary to clarify the role of MAPK15 in ECs, we clearly show that MAPK15 overexpression increases tumorigenicity of human tumor-derived cells and that its depletion is sufficient to constrain cancer cell proliferation and tumor formation in this system. Still, interestingly, high expression of MAPK15 in seminomas (see above) suggests a potential role for this MAPK also in these GCTs. In particular, we are currently interested in understanding whether MAPK15 may act downstream the KIT receptor tyrosine kinase, whose mutation at codon 816 is found in seminomas [58].

In conclusion, our study provides valuable information to understand the molecular mechanisms at the basis of GCT onset and suggests novel approaches to the management of these malignancies. Whether this might be a more general mechanism in other types of cancer rather than a specific feature of the peculiar germ line-derived malignancies warrants further investigation.

## MATERIALS AND METHODS

### Expression vectors and reagents

The pCEFL HA, pCEFL HA MAPK15 [18], pCEFL HA MAPK15_AXXA and pCEFL HA MAPK15_KD [25] expression vectors have been previously described. The corresponding cDNA (MAPK15_WT, MAPK15_AXXA and MAPK15_KD), resistant to siRNA for MAPK15 (siMAPK15-1), were synthesized (GeneART, Thermo Fischer Scientific) with “synonymous” mutations in the sequence recognized by the siRNA and cloned in the pCEFL HA expression vector. The identity and integrity of all vectors was confirmed by DNA sequencing. Cis-platin (Cis-Pt; Sigma Aldrich, Milan, Italy) was used at a final concentration of 1 μM. Bafilomycin A_1_ (Santa Cruz Biotechnology, Heidelberg, Germany) and RO31-8220 (Merck Millipore, Vimodrone, Italy) were used at a final concentration of 100 nM.

### Cell culture and transfections

NTera2/D1 and N2102 embryonic carcinoma cells were obtained from the European Collection of Cell Cultures (ECACC) and maintained in Dulbecco's modified Eagle medium (DMEM) supplemented with 10% fetal bovine serum, 2 mM L-glutamine and 100 units/ml penicillin-streptomycin at 37°C in an atmosphere of 5% CO_2_/air. For western blot and immunoprecipitation experiments, 1×10^6^ NTera2/D1 cells were seeded in 6 cm cell culture dishes and transfected with 1 μg of the each expression vector using Lipofectamine LTX (Life Technologies, Monza, Italy), with addition of Plus Reagent (Life Technologies), according to manufacturer's instructions. Experiments were performed 24 h after transfection. For confocal microscopy experiments, 3×10^5^ NTera2/D1 cells were seeded in 6-well cell culture plates and reverse-transfected with 100 nM of each siRNA using RNAiMax (Life Technologies), according to manufacturer's instructions. For rescue experiments, 24 h after transfection with siRNA, cells were transfected with 0.5 μg of the each expression vector using Lipofectamine LTX (Life Technologies, Monza, Italy), with addition of Plus Reagent (Life Technologies), according to manufacturer's instructions. Forty-eight hours after transfection with siRNA, cells were trypsinized and seeded on coverslips placed onto 12-well cell culture plates at the concentration of 2,5×10^4^ cells/well. Fixation and staining with specific antibodies were performed 72 h after transfection.

In order to generate the NTera2/D1 cell lines stably expressing WT, AXXA and KD isoforms of HA-tagged MAPK15 and the relative negative control, NTera2/D1 cells were transfected with the pCEFL HA, pCEFL HA MAPK15_WT, pCEFL HA MAPK15_AXXA and pCEFL HA MAPK15_KD expression vectors, respectively, and subjected to selection with G-418 sulphate (0.5 mg/ml) for two weeks.

### Knockdown of endogenous MAPK15

MAPK15-specific siRNA #1 (MAPK15-1; target sequence 5′-TTGCTTGGAGGCTACTCCCAA-3′) and control non-silencing siRNA (Scramble, target sequence 5′-AATTCTCCGAACGTGTCACGT-3′) were obtained from Qiagen (Milan, Italy). MAPK15-specific siRNA #2 (MAPK15-2; target sequence 5′- GACAGAUGCCCAGAGAACATT -3′) was obtained from Eurofins Genomics (Ebersberg, Germany). All siRNAs were transfected at a final concentration of 100 nM using RNAiMAX (Life Technologies). Samples were collected 72 h after transfection.

### Antibodies

Primary antibodies used for western blot experiments: phospho-p53 Ser15, cleaved Caspase-3 and cleaved PARP (Cell Signaling, Leiden, The Netherlands), MAPK15 (custom preparation), p53 (Santa Cruz Biotechnology), ACTB (Sigma Aldrich), CDKN1A/p21 (Epitomics, Burlingame, CA), LC3B (Novus Biologicals, Cambridge, UK), SQSTM1/p62 (BD Biosciences, Milan, Italy), GADD45a (Cell Signaling), phospho-ATR (Cell Signaling), phospho-CHK2 (Cell Signaling), PCNA (Cell Signaling). Primary antibodies used for immunofluorescence experiments: γH2A.X (Cell Signaling), 53BP1 (Novus Biologicals) LC3B (MBL, Woburn, MA). Primary antibody used for immunohistochemistry experiments: MAPK15 (custom preparation). Secondary antibodies used for western blot experiments: HRP-conjugated anti-Mouse IgG and HRP-conjugated anti-Rabbit IgG (Santa Cruz Biotechnology). Secondary antibodies used for immunofluorescence experiments: AlexaFluor488-conjugated anti-Rabbit IgG and AlexaFluor555-conjugated anti-Mouse IgG (Life Technologies).

### Western blot analysis

Total lysates were obtained by resuspending cellular pellets in RIPA buffer (50 mM TRIS-HCl pH 8.0, 150 mM NaCl, 0.5% sodium deoxycholate, 0.1% SDS, 1% NP-40) with the addition of protease inhibitors (cOmplete Protease Inhibitor cocktail, EDTA-free; Roche Diagnostics, Monza, Italy) and phosphatase inhibitors (2 mM NaF, 2 mM Na_3_VO_4_; Sigma Aldrich). Total proteins were quantified by Bradford assay and 20-50 μg were used for western blot analysis. 5× Laemmli Loading Buffer (250 mM Tris-HCl pH 6.8, 10% SDS, 50% glycerol, bromophenol blue) was added to the appropriate amount of lysates, which were then heated for 5 min at 95°C. Lysates were loaded on SDS-PAGE poly-acrylamide gel, transferred to Immobilon-P PVDF membrane (Merck Millipore), probed with appropriate antibodies and revealed by enhanced chemiluminescence detection (ECL Plus; GE Healthcare, Milan, Italy). Chemiluminescence signals were digitalized with ImageQuant LAS4000 detection system (GE Healthcare).

### Immunofluorescence (IF), confocal microscopy and intensitometric analysis of fluorescence

NTera2/D1 cells were washed with PBS, fixed with ice-cold methanol for 15 min, and incubated with γH2A.X- or 53BP1-specific primary antibody for 1 h. Alternatively, they were washed with PBS, fixed with 4% paraformaldehyde in PBS for 20 min and permeabilized with 100 μg/ml digitonin (Life Technologies) in PBS for 15 min, and incubated with LC3B-specific primary antibody for 1 h. Cells were then washed three times with PBS, incubated for 30 min with appropriate Alexa Fluor 488-conjugated or Alexa Fluor 555-conjugated secondary antibodies and washed again three times in PBS. Nuclei were stained with 1.5 μM 4′,6-diamidino-2-phenylindole (DAPI; Sigma Aldrich) in PBS for 5 min. Coverslips were mounted in Fluorescence Mounting Medium (Dako, Milan, Italy). Samples were visualized on a TSC SP5 confocal microscope (Leica Microsystems, Milan, Italy) installed on an inverted LEICA DMI 6000CS microscope, using PlanApo 40× 1.25 NA objective or PlanApo 63× 1.4 NA oil immersion objectives. Images were acquired using the LAS AF acquisition software (Leica Microsystems). Fluorescence intensity measurements were performed using the Quantitation Module of Volocity software (Perkin Elmer Life Science, Milan, Italy). At least five representative fields were acquired and analyzed for each sample.

### Measure of oxidative stress

NTera2/D1 were stained with the ROS-specific probe CellROXGreen (Life Technologies), according to manufacturer's instructions. Cells were washed with PBS, fixed with 4% paraformaldehyde in PBS for 20 min and permeabilized with 0.2% Triton X-100 in PBS for 15 min. Nuclei were stained with 1.5 μM DAPI (Sigma Aldrich) in PBS for 5 min. Coverslips were mounted in Fluorescence Mounting Medium (Dako, Milan, Italy). Samples were visualized on a TSC SP5 confocal microscope (Leica Microsystems, Milan, Italy) installed on an inverted LEICA DMI 6000CS microscope, using PlanApo 40× 1.25 NA objective or PlanApo 63× 1.4 NA oil immersion objectives. Images were acquired using the LAS AF acquisition software (Leica Microsystems). Fluorescence intensity measurements were performed using the Quantitation Module of Volocity software (Perkin Elmer Life Science, Milan, Italy). At least five representative fields were acquired and analyzed for each sample.

### Cell count

Cells were seeded onto 6-well plates at 2×10^5^ cells per well and transfected with appropriate siRNA. Viable cells were counted 72 h post transfection in a hemocytometer using the trypan blue dye exclusion assay. All transfections were performed in triplicate and the resulting mean values ± SEM were expressed as a percentage of mean cell number normalized on the Scramble-transfected sample.

### Flow cytometry

Click-iT EdU Flow Cytometry Assay Kit (Life Technologies) was used for cell cycle analysis, according to manufacturer's instructions. Annexin V-PE Apoptosis Detection Kit I (BD Biosciences) was used for the analysis of apoptosis, according to manufacturer's instructions. Samples were acquired on a FACSCanto II flow cytometer (BD Biosciences) equipped with 488-nm solid state and 633-nm HeNe lasers. Data were analyzed with FlowJo software. All analyses were performed in triplicate.

### Analysis of gene expression

Total RNA was purified using Trizol Reagent (Life Technologies). Tissue samples were homogenized using Lysing Matrix Tubes (MP Biomedicals, Santa Ana, CA) and the FastPrep-24 Sample Preparation System (MP Biomedicals) before RNA purification. Reverse transcription was performed with the QuantiTect Reverse Transcription Kit (Qiagen). Real-time PCR (RT-PCR) was performed with the FastStart SYBR Green Master Mix (Roche Diagnostics) on a Rotor-Gene 6000 RT-PCR system (Corbett Life Science, Cambridgeshire, UK). The following primer pairs were used: human MAPK15, 5′-GGAGTTTGGGGACCATCC-3′ and 5′-GCGTTCAGGTCAGTGTCC-3′; mouse MAPK15, 5′-GATCAGAAGCCAGCCAATGT-3′ and 5′-AGGGGTATACCATCGGGAAG-3′; X. laevis MAPK15, 5′-TGGGAAGGGGGCCTATGGAAT-3′ and 5′-TCATTCTGAGCTCGAATCAC-3′; human ACTB, 5′-TGCGTGACATTAAGGAGAAG-3′ and 5′-GCTCGTAGCTCTTCTCCA-3′; human GADD45a, 5′-GGAGGAATTCTCGGCTGGAG-3′ and 5′-CATCTCTGTCGTCGTCCTCG-3′; human 14-3-3σ, 5′-ACAACCTGACACTGTGGACG-3′ and 5′-CCTTTGGAGCAAGAACAGCG-3′.

### In-situ hybridization (ISH)

Tissues were fixed O/N by immersion in modified Davidson's fluid (30% of a 37–40% solution of formaldehyde, 15% ethanol, 5% glacial acetic acid, 50% DEPC-treated distilled H_2_O) [59] and embedded in paraffin by standard procedures. For *in-situ* hybridization analysis, testis sections (8 μm) mounted on positively charged microscope slides were deparaffinized and rehydrated, washed three times in 1× phosphate buffered saline (PBS, 137 mM NaCl, 2.7 mM KCl, 1.5 mM KH_2_PO_4_, 7.3 mM Na_2_HPO_4_), digested using 10 μg/ml proteinase K in 1× PBS for 15 min at 37°C, washed again twice in 1× PBS and post-fixed with 4% paraformaldehyde for 5 min. Samples were acetylated with a solution of 1,2% triethanolamine, 0,0018N HCl, 0,25% acetic anhydride in H_2_O. Sections were prehybridized with Hybridization Buffer (5× SSC, 50% formamide, 1× Denhardt's Solution, 9.2 mM citric acid, 50 μg/ml heparin, 250 μg/ml yeast RNA in H_2_O) for 3h and incubated with 25 nM DIG-labeled LNA probe (Exiqon, Vedbaek, Denmark) in Hybridization Buffer O/N at hybridization temperature (55°C) in a humidified horizontal chamber (5× SSC, 50% formamide in H_2_O). Three post-hybridization washes were performed in 0.1× SSC for 1h at 60°C. Sections were incubated with Blocking Buffer (0.5% Blocking Reagent (Roche Applied Science), 50 mM Tris-HCl pH 7.5, 5 mM EDTA) for 2h at room temperature and with an anti-DIG FAb antibody conjugated to alkaline phosphatase (Roche Diagnostics) overnight at 4°C in a humidified horizontal chamber (PBS). Section were then washed three times with Wash Buffer A (100 mM Tris-HCl pH 7.5, 150 mM NaCl) for 5 min and once in Wash Buffer B (100 mM Tris-HCl pH 9.5, 100 mM NaCl, 50 mM MgCl_2_) for 10 min. Staining was visualized by developing sections with NBT/BCIP solution (Roche Diagnostics) in a humidified chamber protected from light. Sections were mounted with 90% glycerol and imaged with an inverted LEICA DMI 3000 microscope equipped with a 40× dry HCX PL FLUOTAR 0.60 NA objective and a DFC31DFX camera (Leica Microsystems). The following probes were used: mouse *Mapk15*, 5′-DigN/TGCGAACTTCCACATGCTCA-3′; sense miR159 from Arabidopsis thaliana, 5′-DigN/AGAGCTCCCTTCAATCCAAA-3′; *Actb*, 5′-DigN/CTCATTGTAGAAGGTGTGGTGCCA-3′; *U6 snRNA*, 5′-DigN/CACGAATTTGCGTGTCATCCTT-3′.

### Immunohistochemistry (IHC)

Tissues were fixed by immersion in 10% formalin and paraffin-embedded according to standard procedures. Two 4 μm-thick serial sections were cut and mounted on poly-L-lysine-coated glass slides. Sections were deparaffinized and underwent antigen retrieval by microwave oven treatment (5 min × 3 times, in 1% sodium citrate buffer, pH 6.0). Non-specific bindings were blocked by incubation (30 minutes at room temperature) with 1.5 % non-immune mouse serum (Dako). Endogenous peroxidases were quenched with 0.3% hydrogen peroxide in methanol. Slides were rinsed twice with Tris-HCl buffer and incubated 1 hour at room temperature with anti-MAPK15 antibody. For negative controls, non-immune serum in TBS buffer (1:500) was used instead of the primary antibody.

The standard streptavidin-biotin linked horseradish peroxidase (LSAB) technique was then performed with the Dako LSAB kit HRP (Dako). 3,3′-diaminobenzidine (DAB; Vector Laboratories, Burlingame, CA) was used as chromogenic substrate. Sections were counterstained with Mayer's haematoxylin for 30s, mounted and cover-slipped with a synthetic medium.

The immunohistochemical expression was evaluated semiquantitatively and cells were classified as low staining, [from 0 (<5%), to + (5-25%)], and high staining [from ++ (26-50%) to +++ (>50%)].

### Xenografts

All protocols involving animals have been approved by the internal Animal Welfare Body and by Italian Ministero della Salute. For each injection, 2×10^6^ NTera2/D1 cells were resuspended in 100μl of 50% Matrigel (BD Biosciences) in PBS. Six-week-old athymic nude-Fox1^nu^ female mice (Harlan Laboratories, Correzzana, Italy) were injected in both flanks using a 21-gauge needle. The mice were anesthetized with isoflurane throughout the procedure. Five mice were injected on both flanks, for a total of 10 injections. The animals were then monitored for tumor growth and tumor size was measured twice a week with a caliper (2Biological Instruments, Besozzo, Italy). Tumor volumes were calculated using the formula *V*=*W* ^2^×*L*×0.5, where *W* and *L* are tumor width and length, respectively. The mice were sacrificed by CO_2_ inhalation under sedation when tumors reached a volume of approximately 1000 mm^3^. At the endpoint, xenograft were excised from sacrificed mice, suspended in RIPA buffer with the addition of protease inhibitors and phosphatase inhibitors and homogenized in lysing matrix tubes (MP biomedicals, Eschwege, Germany) using a FastPrep-24 homogenizer (MP biomedicals).

### Statistical analysis

Significance (p value) was assessed by Student's t test, using GraphPad Prism6 software. Asterisks were attributed as follows: *p<0.05, **p<0.01, ***p<0.001, ****p<0.0001.

## SUPPLEMENTARY FIGURES


