# Supplementary material for: MAPK15 upregulation promotes cell proliferation and prevents DNA damage in male germ cell tumors
Source: Oncotarget. 2016 Mar 14;7(15):20981–98. doi: 10.18632/oncotarget.8044 (PMC4991506; doi:10.18632/oncotarget.8044)
Supplement: Supplementary file 1 [file oncotarget-07-20981-s001.pdf]

SUPPLEMENTARY FIGURES

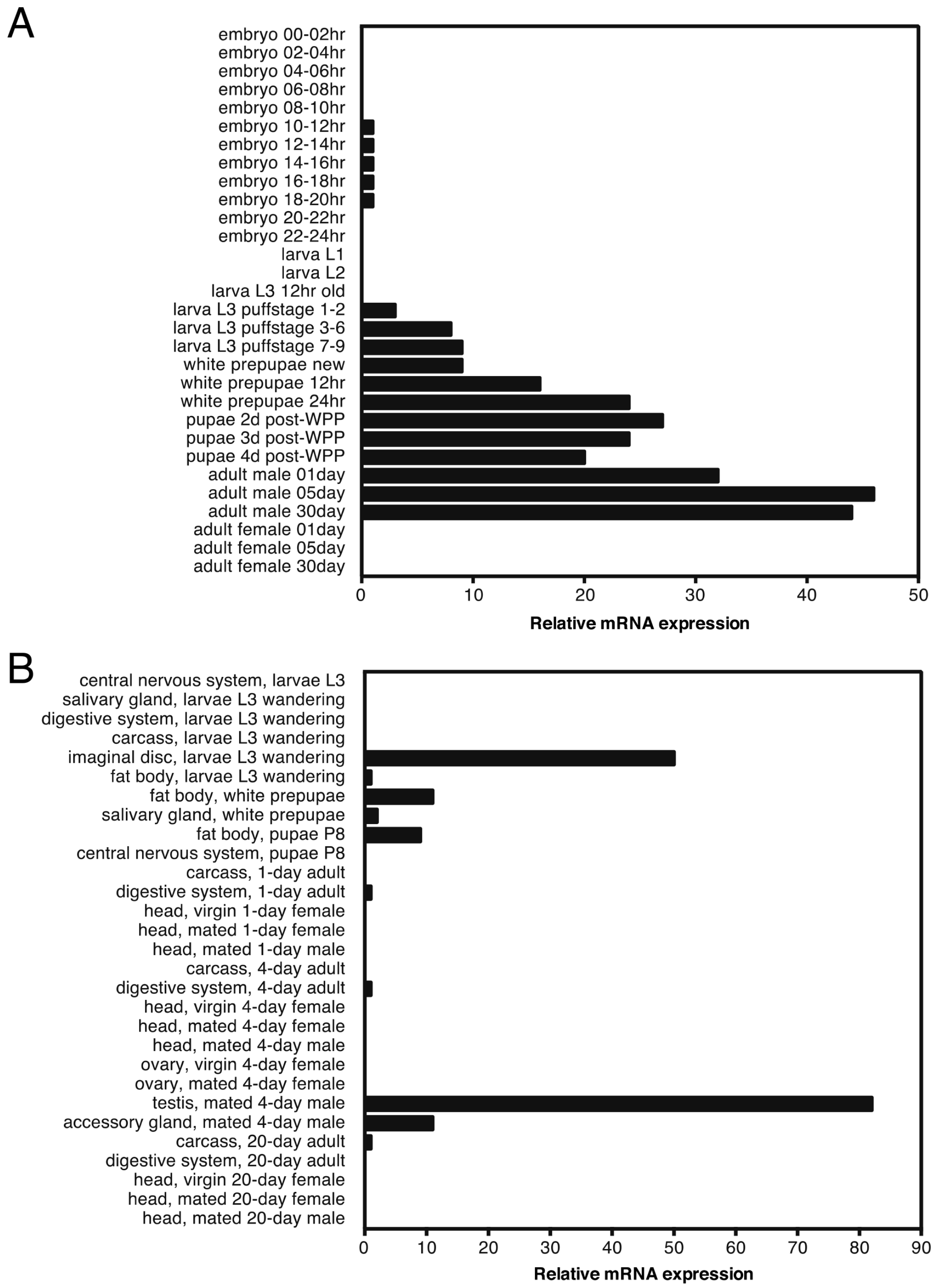

Supplementary Figure 1: Elevated expression of MAPK15 in male gonads is a conserved trait in D. melanogaster. **A.** Analysis of the expression of CG31703, the MAPK15 ortholog in Drosophila, at different developmental stages. **B.** Analysis of the expression of CG31703 in different tissues. Data were obtained from the public access on-line database FlyBase (http://flybase.org).

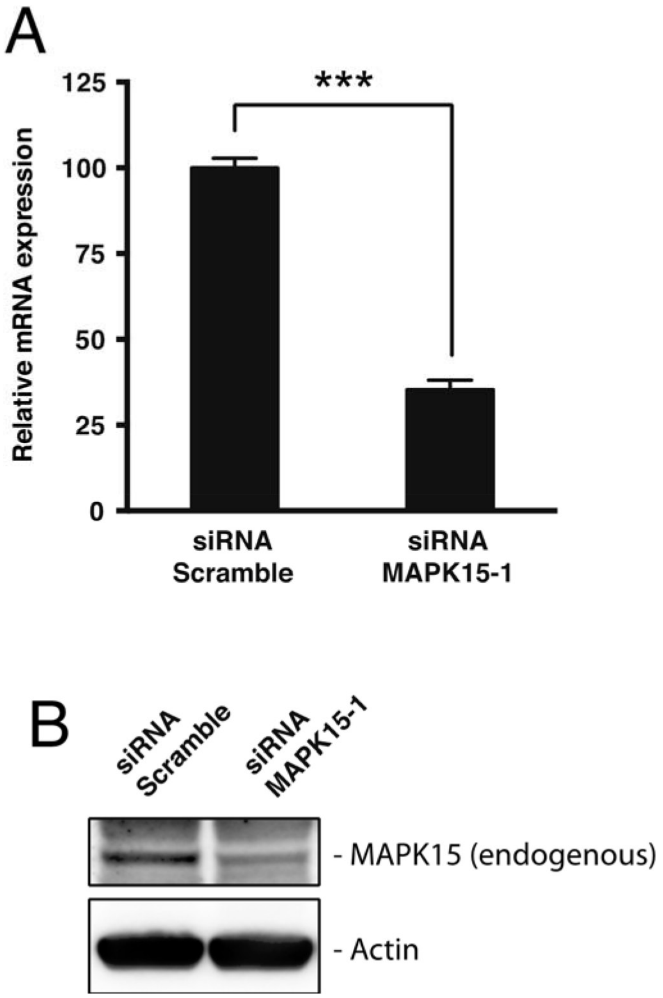

Supplementary Figure 2: MAPK15 RNA interference. Expression levels of MAPK15 in NTera2/D1 cells following RNA interference with siRNA specific for MAPK15, compared to a control transfected with non-silencing (Scramble) siRNA, as assessed by quantitative real-time PCR 72 hrs post-transfection **(A)** and by anti-MAPK15 western blot **(B)**. Each bar represents the average ± SEM of three PCR replicates. Significance (p value) was assessed by Student's t test. Asterisks were attributed as follows: ***$p<0.001$.

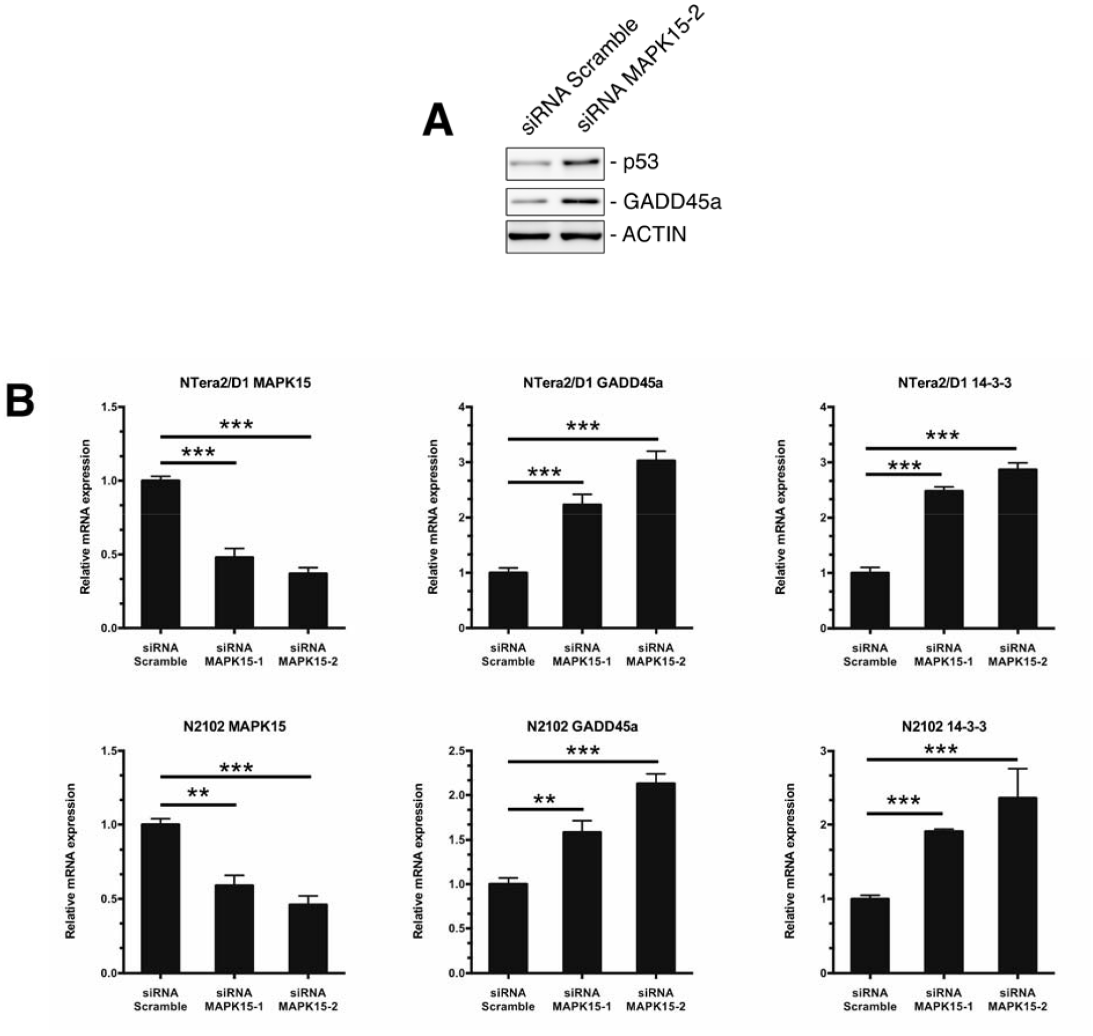

Supplementary Figure 3: Confirmation of results obtained with alternative MAPK15 siRNAs and NTera2/D1 cells. **A.** Lysates from NTera2/D1 cells interfered for MAPK15 (siMAPK15-2), compared to a control transfected with Scramble siRNA, were probed with antibodies specific for GADD45a. ACTB (actin) was used as loading control and for normalization purposes. **B.** Expression levels of *MAPK15*, *GADD45a* and *14-3-3* in NTera2/D1 and N2102 cells following RNA interference with siRNA specific for MAPK15, compared to a control transfected with non-silencing (Scramble) siRNA, as assessed by quantitative real-time PCR 72 h post-transfection. Significance (p value) was assessed by Student's t test. Asterisks were attributed as follows: $**p<0.01$, $***p<0.001$. Each bar represents the average ± SEM of three PCR replicates.

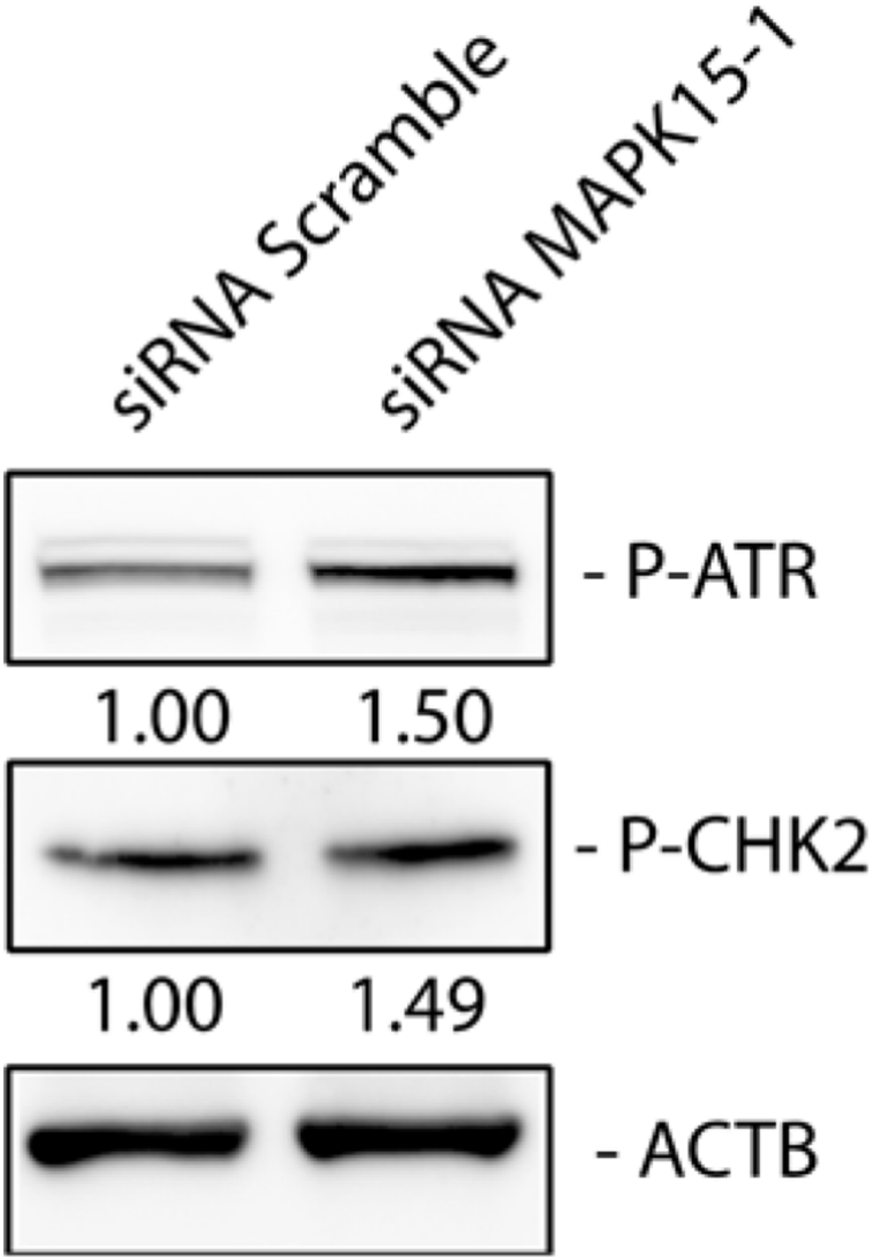

Supplementary Figure 4: Lysates from NTera2/D1 cells interfered for MAPK15 (siMAPK15-1), compared to a control transfected with Scramble siRNA, were probed with antibodies specific for phospho-ATR and phospho-CHK2. ACTB (actin) was used as loading control and for normalization purposes. Intensitometric analysis was performed with ImageQuant TL software (GE Healthcare).

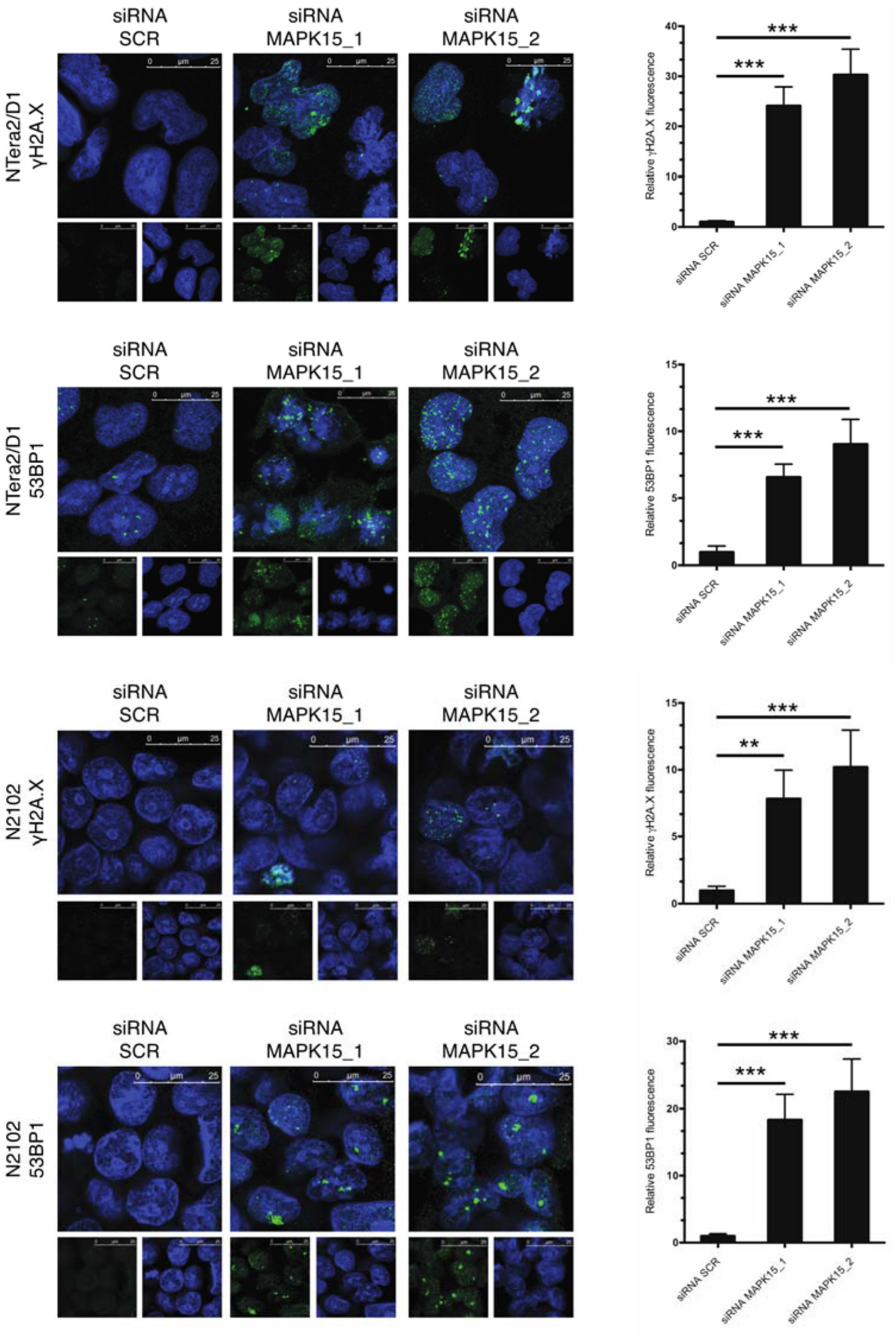

Supplementary Figure 5: MAPK15 protects the genomic integrity of human GCT-derived cell lines. Representative confocal microscopy images showing DNA damage, scored as accumulation of nuclear γH2A.X and 53BP1 foci, in NTera2/D1 and N2102, cells following transfection with non-silencing (Scramble) siRNA (SCR) or siRNA specific for MAPK15 (MAPK15-1 and MAPK15-2). Cells were stained with anti-γH2A.X and 53BP1 primary antibodies and Alexa Fluor 488-conjugated secondary antibody. Nuclei were stained with DAPI. Intensitometric analysis of nuclear γH2A.X and 53BP1 fluorescence, identified as green fluorescence colocalizing with DAPI. Total nuclear green fluorescent signal was quantified in five representative fields using Volocity software. A total of approximately 100 cells were analyzed for each sample. Significance (p value) was assessed by Student's t test. Asterisks were attributed as follows: **$p<0.01$, ***$p<0.001$.

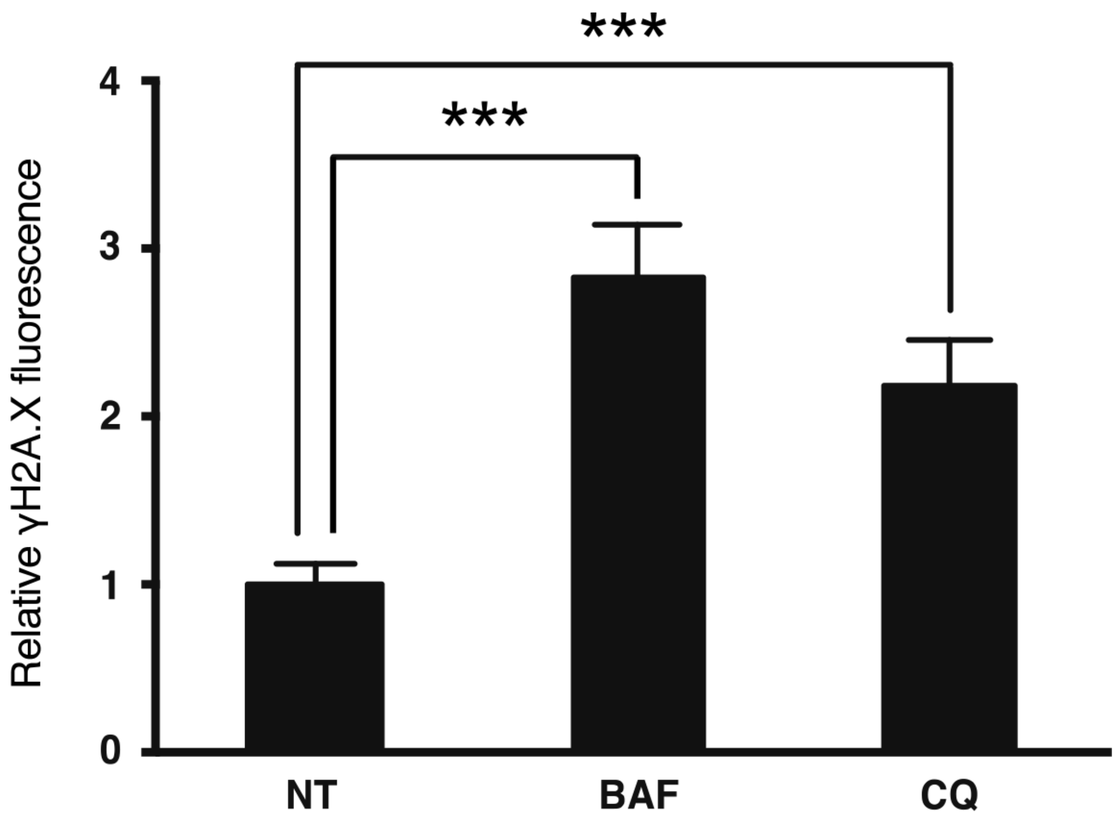

Supplementary Figure 6: Pharmacological inhibition of autophagy in NTera2/D1 cells increases DNA damage, scored as accumulation of nuclear γH2A.X foci, following treatment for 24 h with 100 nM bafilomycin A1 (BAF) or 10 µM cloroquine (CQ), compared to vehicle (NT). Cells were stained with anti-γH2A.X primary antibody and Alexa Fluor 488-conjugated secondary antibody. Nuclei were stained with DAPI. Nuclear γH2A.X fluorescence was identified as green fluorescence colocalizing with DAPI. Total nuclear green fluorescent signal was quantified in five representative fields using Volocity software. Bars represent the average ± SEM of five representative microscopy fields. Significance (p value) was assessed by Student's t test. Asterisks were attributed as follows: ***$p<0.001$.

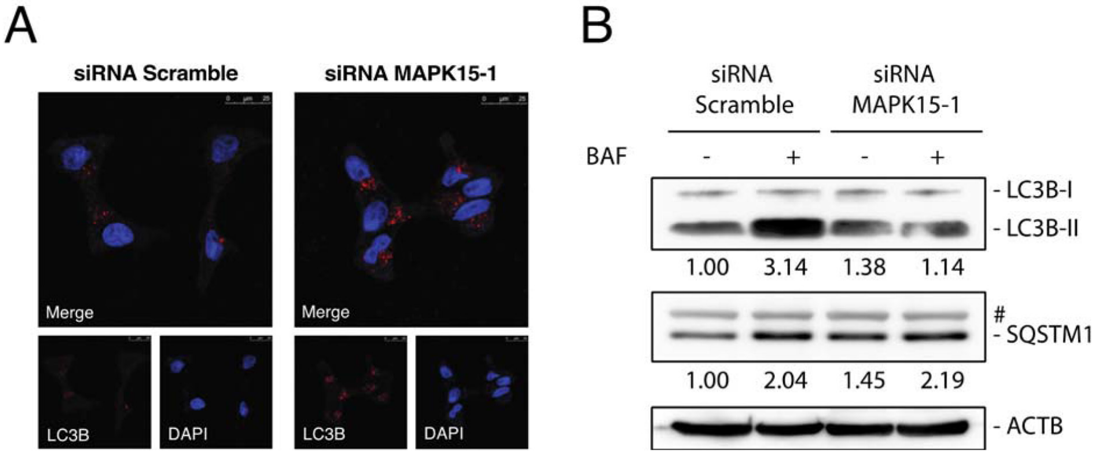

Supplementary Figure 7: MAPK15 induces autophagy in human GCT-derived cell lines. A. Representative confocal microscopy images showing accumulation of LC3B-positive autophagic vesicles, in NTera2/D1 cells following transfection with non-silencing (Scramble) siRNA (left panel) or siRNA specific for MAPK15 (right panel). Cells were stained with anti-LC3B primary antibody and Alexa Fluor 555-conjugated secondary antibody. Nuclei were stained with DAPI. **B.** Autophagic flux in NTera2/D1 cells following transfection with Scramble siRNA or siRNA specific for MAPK15 in presence of 100 nM bafilomycin A1 (BAF). Lysates were probed with antibodies specific for LC3B and SQSTM1/p62. ACTB (actin) was used as loading control and for normalization purposes. Intensitometric analysis was performed with ImageQuant TL software (GE Healthcare). # refers to an aspecific band.

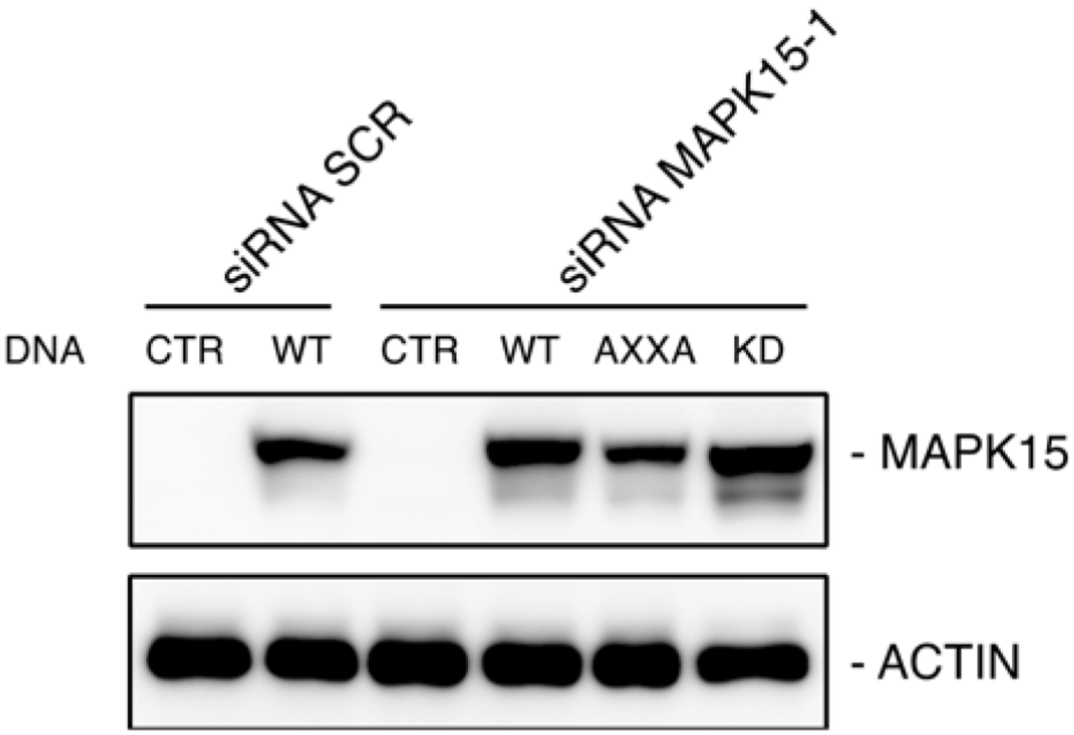

Supplementary Figure 8: Ectopic expression of wild-type (WT), Y340A-I343A autophagy-deficient (AXXA), and kinase-dead (KD) MAPK15 following MAPK15 knockdown with specific siRNA. Control sample (CTR) is transfected with the empty vector. Lysates were probed with an antibody specific for MAPK15. ACTB (actin) was used as loading control.

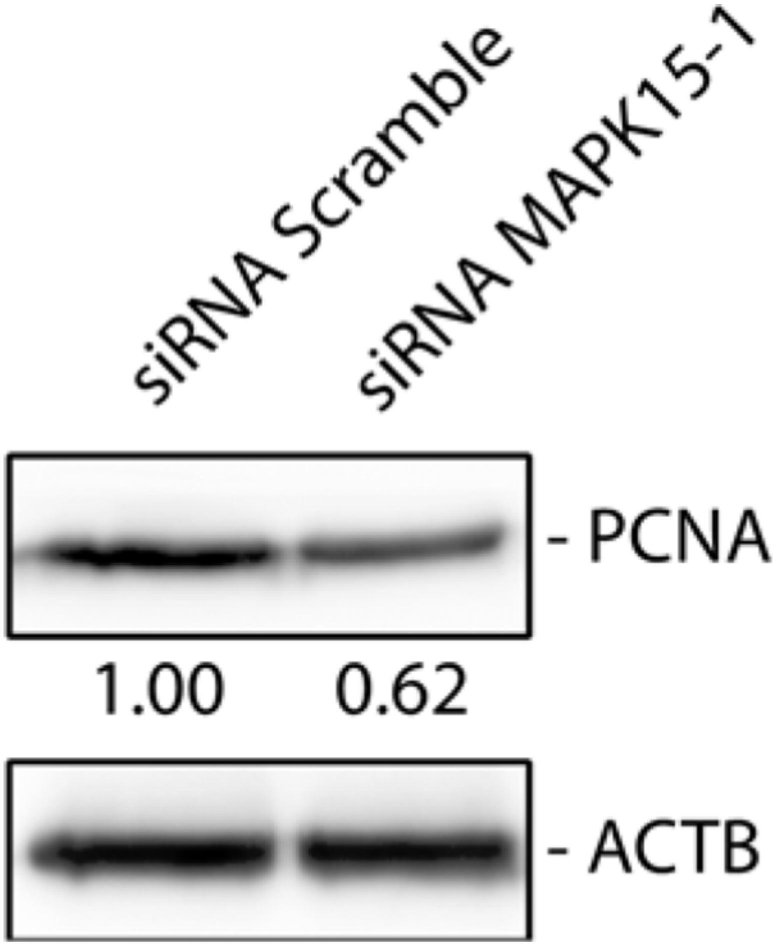

Supplementary Figure 9: MAPK15 controls PCNA protein levels. Lysates from NTera2/D1 cells interfered for MAPK15 (siMAPK15-1), compared to a control transfected with Scramble siRNA, were probed with antibodies specific for PCNA. ACTB (actin) was used as loading control and for normalization purposes. Intensitometric analysis was performed with ImageQuant TL software (GE Healthcare).
